# Detecting Signs of Depression in Tweets in Spanish: Behavioral and Linguistic Analysis

**DOI:** 10.2196/14199

**Published:** 2019-06-27

**Authors:** Angela Leis, Francesco Ronzano, Miguel A Mayer, Laura I Furlong, Ferran Sanz

**Affiliations:** 1 Research Programme on Biomedical Informatics Hospital del Mar Medical Research Institute Department of Experimental and Health Sciences, Universitat Pompeu Fabra Barcelona Spain

**Keywords:** depression, social media, mental health, text mining

## Abstract

**Background:**

Mental disorders have become a major concern in public health, and they are one of the main causes of the overall disease burden worldwide. Social media platforms allow us to observe the activities, thoughts, and feelings of people’s daily lives, including those of patients suffering from mental disorders. There are studies that have analyzed the influence of mental disorders, including depression, in the behavior of social media users, but they have been usually focused on messages written in English.

**Objective:**

The study aimed to identify the linguistic features of tweets in Spanish and the behavioral patterns of Twitter users who generate them, which could suggest signs of depression.

**Methods:**

This study was developed in 2 steps. In the first step, the selection of users and the compilation of tweets were performed. A total of 3 datasets of tweets were created, a depressive users dataset (made up of the timeline of 90 users who explicitly mentioned that they suffer from depression), a depressive tweets dataset (a manual selection of tweets from the previous users, which included expressions indicative of depression), and a control dataset (made up of the timeline of 450 randomly selected users). In the second step, the comparison and analysis of the 3 datasets of tweets were carried out.

**Results:**

In comparison with the control dataset, the depressive users are less active in posting tweets, doing it more frequently between 23:00 and 6:00 (*P*<.001). The percentage of nouns used by the control dataset almost doubles that of the depressive users (*P*<.001). By contrast, the use of verbs is more common in the depressive users dataset (*P*<.001). The first-person singular pronoun was by far the most used in the depressive users dataset (80%), and the first- and the second-person plural pronouns were the least frequent (0.4% in both cases), this distribution being different from that of the control dataset (*P*<.001). Emotions related to sadness, anger, and disgust were more common in the depressive users and depressive tweets datasets, with significant differences when comparing these datasets with the control dataset (*P*<.001). As for negation words, they were detected in 34% and 46% of tweets in among depressive users and in depressive tweets, respectively, which are significantly different from the control dataset (*P*<.001). Negative polarity was more frequent in the depressive users (54%) and depressive tweets (65%) datasets than in the control dataset (43.5%; *P*<.001).

**Conclusions:**

Twitter users who are potentially suffering from depression modify the general characteristics of their language and the way they interact on social media. On the basis of these changes, these users can be monitored and supported, thus introducing new opportunities for studying depression and providing additional health care services to people with this disorder.

## Introduction

### Background

Mental health is an essential component of our health. The World Health Organization (WHO) defines mental health as a “state of well-being in which people realize their potential, cope with the normal stresses of life, work productively, and contribute to their communities” [1]. Good mental health is about being cognitive, emotionally and socially healthy and it helps to determine the way we think and feel, in relation with others and how we make choices. Several factors, such as genetic, sociocultural, economic, political and environmental aspects, shape and influence our mental health. In the last few years, mental disorders have become a major concern in public health, and they are one of the main causes of the overall disease burden worldwide. They have devastating consequences for both patients and their families [2-7]. According to the WHO, depressive disorders are the most common among the mental illnesses [8]. Such disorders conditions are characterized by sadness, loss of interest and pleasure, feelings of guilt or low self-worth, disturbed sleep or appetite, feelings of tiredness, and poor concentration [8]. In 2018, at the global level, more than 300 million people were suffering from depression, and it is the main contributor to global disability. Depression has several consequences, both personal and social costs [9,10]. In some cases, depression can lead to suicide ideation and attempts [2,11]. The prevalence of this disorder changes depending on age, but it affects the whole population, from children and adolescents to elderly people. From 2005 to 2015, the number of people with depression increased by around 18% [12]. In this context, social media platforms allow to observe the activities, thoughts, and feelings of people’s daily lives and thereby investigate their emotional well-being. This domain has become a new growing area of interest in public health and health care research [13-16]. People with depression often use social media to talk about their illness and treatment, share information and experiences, seek social support and advice, reduce social isolation, and manage their mental illness [15-21]. In addition, access to mobile devices facilitates the use of social media platforms, such as Twitter and Facebook, at any time and at any place. Social media, such as Twitter, is by nature social, and we can consequently find social patterns in Twitter feeds, thereby revealing key aspects of mental and affective disorders [22]. Social media has become an important source of health-related information, which allows us to detect and predict affective disorders and which can be used as an additional tool for mental health monitoring and infoveillance [23-26]. Furthermore, the application of different methodologies based on natural language processing and machine learning technologies has proved to be effective in supporting and automating the identification of early signs of mental illness by analyzing the content shared on the Web by individuals [13-15,27]. This human interaction with social media contributes to build the so-called digital phenotype, reshaping disease expression in terms of the lived experience of individuals and detecting early manifestations of several conditions [28]. Twitter is an internet microblogging social media service that allows users to post short messages about facts, feelings and opinions, and, as shown in previous studies, users’ health conditions [15]. Twitter is one of the most important social media platforms in terms of number of users, with more than 330 million active users worldwide [29]. Since November 2017, the maximum number of characters of a tweet has been increased from 140 to 280. By analyzing huge amounts of text, researchers can link everyday language use with social behavior and personality [30,31]. Language, as a means of communication, constitutes an essential element for providing valuable insights about people’s interests, feelings and concerns [32]. For this reason, the analysis of the messages posted on social media platforms may provide information about many personality traits, lifestyles, and psychological disorders [13,33,34]. The potential anonymity of social media encourages its users to be more willing to report health information, such as details of their mental disorders and treatments received. In addition, it is seen as a way to communicate and receive support from others with similar experiences, avoiding the isolation and fighting the social stigma of these conditions [12,15,17,19,32,35]. Nevertheless, users suffering from depression may also feel uncomfortable socializing and consuming information on social media platforms [36]. Several features of the messages, such as number and frequency of tweets, distribution throughout the day or during the night hours, and their seasonal character, can be used for the detection and monitoring of mental disorders, such as depression [20]. This knowledge can help health care professionals and health institutions and services in the decision-making processes to ensure better management of patients suffering from depression.

### Objectives

There are many studies that have used data mining and machine learning techniques on social media platforms to automatically identify people with mental health problems, such as depression, posttraumatic stress disorder, schizophrenia, or eating disorders, usually focusing the studies on messages written in English [20,37-39]. As far as we know, on social media, there are no studies about mental disorders that analyze messages written in Spanish. Taking into account that Spanish speaking countries, such as Spain and Mexico, are among the 10 most active Twitter users in the world, with more than 6 million and 7 million users, respectively [40], we focused our research on the expression of depression in Spanish language tweets. The aim of this study was to identify the linguistic features of tweets written in Spanish and the behavioral patterns of the corresponding Twitter users that could suggest signs of depression.

## Methods

### Study Steps

This study was designed and developed in 2 steps, with the aim of analyzing the linguistic patterns and behavioral features of Twitter users suffering from depression in comparison with the general population of Twitter users. The study was focused on tweets written in Spanish. In the first step, the selection of users and the compilation of tweets were performed. Given the design and purpose of the study, we decided to use the Twitter Application Programming Interface (API) [41]. Using this API, 3 datasets of tweets were created:

1. The *depressive users* dataset was made up of the timeline of 90 users who publicly mentioned on their Twitter profile that they suffer from depression.
2. The *control* dataset was made up of the timeline of 450 randomly selected Twitter users.
3. The *depressive tweets* dataset was constituted by a manual selection of tweets from the depressive users dataset, which specifically included expressions indicative of depression.

**Figure 1:**
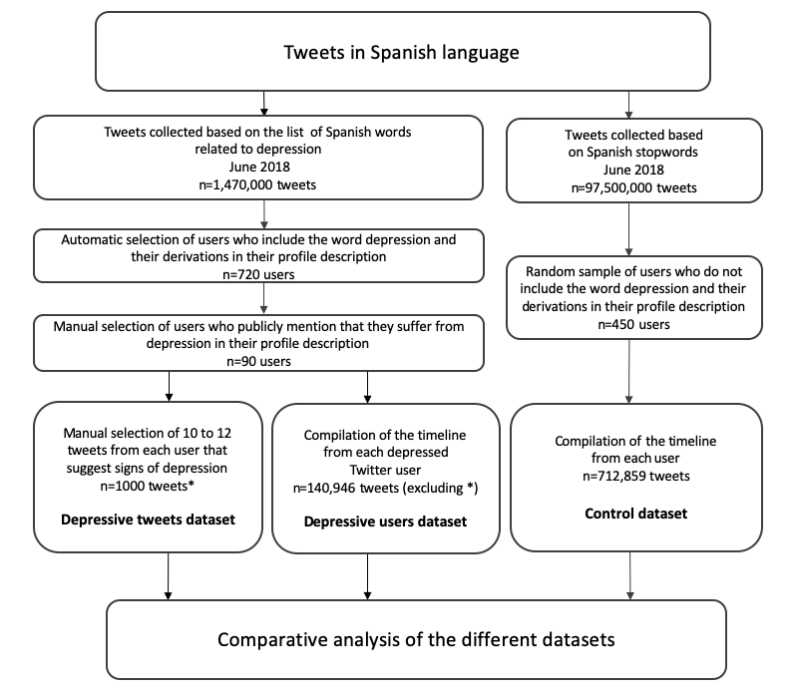
Flow diagram of the study process.

In the second step, comparison and analysis of the 3 datasets of tweets (control, depressive users, and depressive tweets datasets) were carried out to spot their distinguishing features. In the rest of this section, we will describe the methodology in detail. The flow diagram of the study is depicted in Figure 1.

### Data Collection and User Selection

The selection of the tweets and their users was based on the filtered real-time streaming support provided by the Twitter API. In the first step, we selected the users who showed potential signs of depression on Twitter on the basis of the 20 most frequent words in Spanish expressed by patients suffering from depression in clinical settings. These words were jointly identified and selected by a psychologist and a family physician with clinical experience and were based on the definition and general features of depression according to the Diagnostic and Statistical Manual of Mental Disorders [42]. The list of words used and their English translations are shown in Textbox 1.

**List of Spanish words related to depression and their English translations.**

- agobiado/a (overwhelmed)
- agotado/a (exhausted)
- angustiado/a (distressed)
- ansiedad (anxiety)
- ansioso/a (anxious)
- cansado/a (tired)
- decaído (low)
- depresión (depression)
- depresivo/a (depressed as a condition)
- deprimido/a (depressed as state)
- desanimado/a (discouraged)
- desesperado/a (desperate)
- desmotivado/a (demotivated)
- insomnio (insomnia)
- llorar (cry)
- nervioso (nervous)
- preocupado/a (worried)
- solo/a (lonely)
- triste (sad)
- vacío/a (empty)

During June 2018, 1,470,000 tweets, including 1 or more occurrences of the words listed in Textbox 1, were collected. From this collection of tweets and to select the users who publicly stated in the textual description associated to their profile that they suffered from depression, all the profile descriptions, including 1 or more occurrences of the word “depr” and all the possible derivations related to the word depression in Spanish, such as “depre,” “depresión,” “depresivo,” “depresiva,” “deprimido,” and “deprimida,” were considered. From the 720 users who included 1 or more of these words in their description profile, 90 users who stated they suffered from depression or were receiving treatment for depression were selected for the analysis. This selection was performed by a psychologist, verifying that the statements were related to real expressions of depression, excluding quotes, jokes, or fake ones. For each of these depressed Twitter users, we collected all the most recent tweets from their timeline, up to a maximum of about 3200 tweets. Thus, a total of 189,669 tweets were collected, a figure that was reduced to 140,946 after discarding the retweets. These 140,946 tweets constituted the *depressive users dataset*. Examples of sentences appearing in the user profiles that were used for selecting the depressive users are:

- “Paciente psiquiátrico con depresión crónica” (*Psychiatric patient with chronic depression;* example of a profile sentence that indicates depression).
- “Colecciono errores traducidos a tweets depresivos y a uno que otro impulso de amor” (*I gather errors translated into depressing tweets and into one or another love impulse*; example of a profile sentence that does not indicate depression).

Once the users with profile sentences indicating depression had been retrieved, their Twitter timelines were collected. Only those users having in their timeline at least 10 tweets that suggested signs of depression were retained for further analyses. For each user, the selection of these tweets was performed by manually inspecting the tweets of the user’s complete timeline in reverse temporal order, starting from the most recent one to the oldest tweet of the timeline retrieved by means of the Twitter API *.* Finally, a total number of 1000 tweets issued by the 90 depressive users, suggesting signs of depression, were detected and used for the analysis. This set of tweets provided us with the *depressive tweets dataset*, which was used to analyze linguistic features of tweets showing signs of depression. It has to be mentioned that these 1000 tweets were not to be included in the depressive users dataset (see Figure 1). At the same time, more than 97,500,000 tweets were also collected in June 2018: such tweets were gathered by listening to the public Twitter stream during this time span by only considering tweets with Spanish textual contents (as detected by Twitter language identification support).

Given that Twitter requires more restrictive filters than just the language of the tweets, we used a list of the most frequently used Spanish words (stopwords) to retrieve all tweets that included 1 or more of these words. The vast majority of Spanish tweets should match this criterion. A sample of 450 users who did not mention in their profile the word depression and its derivations were selected randomly from the 97,500,000 tweets. The complete timelines of these users were compiled (1,141,021 tweets), which were reduced to 712,589 once retweets were removed. These 712,589 tweets constituted the *control dataset*. To identify the language of a tweet, we relied on the language automatically identified by Twitter for each tweet, selecting tweets in Spanish. It has to be noted that these data can contain some tweets from unidentified depressive users.

### Data Analysis

A comparison of the 3 datasets was performed to determine the existence of differential linguistic and behavioral features. The different features that were analyzed are shown in Table 1.

The textual content of each tweet was analyzed by means of the following sequence of steps:

- Tokenization performed by means of a custom Twitter tokenizer included in the Natural Language Toolkit [43].
- Part-of-Speech (POS) tagging performed by means of the Freeling Natural Language Processing tool in order to analyse the usage patterns of grammatical categories (eg, adjectives, nouns, or pronouns) in the text of tweets [44].
- Identification of negations performed by relying on a custom list of Spanish negation expressions, such as *nada* (nothing), *nadie* (nobody), *no* (no), *nunca* (never), and alike.
- Identification of occurrences of positive and negative words inside the text of each tweet by means of 2 Spanish polarity lexicons: the Spanish Sentiment Lexicon and the Spanish SentiCon Lexicon [45,46]. We exploited 2 lexicons to consider and compare 2 approaches of modeling polarity in Spanish texts, thus reducing any language modeling bias that the use of a single language resource could introduce.
- Identification of words and expressions associated to the basic emotions [47] by using the Spanish Emotion Lexicon [48]. Such emotions are *alegría* (happiness), *enojo* (anger), *miedo* (fear), *repulsión* (disgust), *sorpresa* (surprise), and *tristeza* (sadness).

All the tools and aforementioned resources are publicly available. The statistical analyses were carried out with the R version 3.4.3 (R Development Core Team) and SPSS Statistics version 23.0 (IBM), applying the relevant test for each type of comparison to be carried out.

**Table 1:** Characteristics of the tweets analyzed.

Feature	Analyses performed
Distributions over time	Tweets throughout the day (per hour) Tweets throughout the week
Part-of-Speech	Number of words by grammatical categories (part-of-speech tags) Number of personal pronouns
Counts	Number of characters 200 most frequent words (word cloud) Number of hashtags, links, mentions, and emojis
Emotion analysis	Emotion types and frequencies
Negations	Negation words types and frequencies
Polarity analyses	Polarity of tweets on the basis of Spanish Sentiment Lexicon and Spanish SentiCon Polarity

### Ethical Approval

The protocol used in this study was approved by the Ethics Committee of Parc Salut Mar (approval number 2017/7234/1).

## Results

### Distribution Over Time

**Figure 2:**
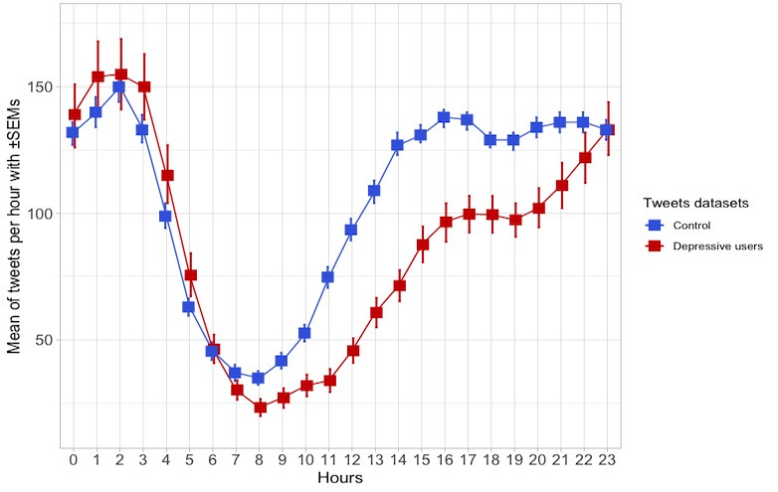
Number of tweets and retweets per hour of the control and depressive users datasets (mean±standard error of mean). SEM: standard error of mean.

**Figure 3:**
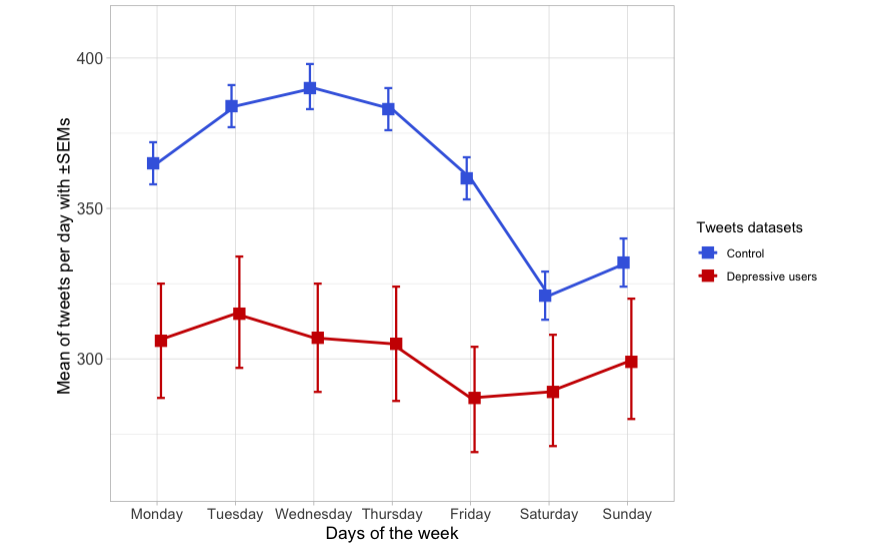
Number of tweets and retweets throughout the week of the control and depressive users datasets (mean±standard error of mean). SEM: standard error of mean.

Regarding the distribution of tweets over time, the number of tweets per hour and throughout the week of control and depressive users datasets were compared. The tweet hours were adjusted by the user’s time zone. As shown in Figure 2, the depressive users are less active in generating tweets than the control ones, reaching both groups the same activity level between 23:00 and 6:00. The comparison of the temporal distributions of tweets between both datasets was carried out by means of a repeated measures analysis of variance (Greenhouse-Geisser F=6.605; *P*<.001). As shown in Figure 3, the activity throughout the week of the depressive users dataset presented more regular activity than the control dataset, whose users’ activity showed a sharp drop during the weekend. The differences between these datasets were statistically significant (Greenhouse-Geisser F=4.153; *P*=.008).

### Part-of-Speech

As for the analysis of POS corresponding to the number of words by grammatical categories in each tweet, we compared the 3 datasets of tweets: the control, depressive users, and depressive tweets datasets. As previously stated, the tweets of the depressive tweets dataset were removed from the depressive users dataset. The frequencies of words in each group are shown in Table 2. The number of nouns used in the control group almost doubles that of the depressive users dataset. By contrast, verbs are more frequently used in the depressive users dataset than in the control dataset. There were statistically significant differences between the control and the depressive users datasets (χ^2^_7_=1,242,600; *P*<.001), between the control and the depressive tweets datasets (χ^2^_7_=2,105.7; *P*<.001), and between the depressive users and the depressive tweets datasets (χ^2^_7_=15,888; *P*<.001).

**Figure 4:**
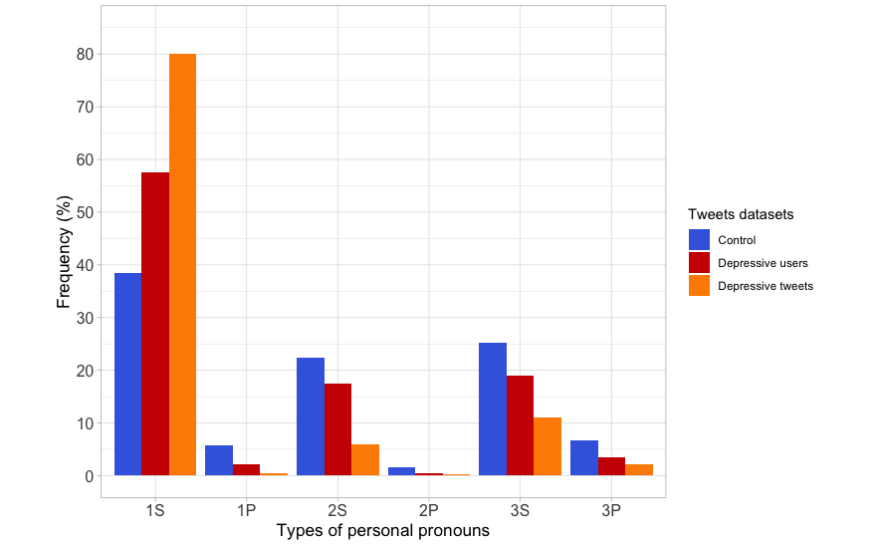
Frequency of the different types of personal pronouns in the control, depressive users, and depressive tweets datasets. 1S: first-person singular; 1P: first-person plural; 2S: second-person singular; 2P: second-person plural; 3S: third-person singular; 3P: third-person plural.

In relation to the different types of pronouns in the control dataset, we detected 396,181 personal pronouns (51.38%; 396,181/770,955), the first-person singular (38.37%; 152,013/396,181) being the most used. A similar profile was observed in the depressive users dataset, where 124,614 pronouns were found (55.16%; 124,614/225,913), the first-person singular remaining the most used (57.59%; 71,768/124,614). In the depressive tweets dataset, 865 personal pronouns (53.16%; 865/1,627) were identified, and the first-person singular pronoun was by far the most used (80.00%; 692/865). The frequencies of personal pronouns in the different datasets are shown in Figure 4. There were statistically significant differences between the control and the depressive users datasets (χ^2^_5_=15,912; *P*<.001), between the control and the depressive tweets datasets (χ^2^_5_=638.7; *P*<.001), and between the depressive users and the depressive tweets datasets (χ^2^_5_=183.9; *P*<.001).

In relation to the number of characters per tweet, the mean of characters per tweet in the control and depressive users datasets was 83.48 (SD 40.57) and 65.76 (SD 36.99) characters, respectively, with statistically significant differences between them (*t*_213770_=161.6; *P*<.001). On the other hand, the mean in the depressive tweets dataset was 67.51 (SD 38.28), which was not statistically significant and different in comparison with the depressive dataset (*t*_1012.3_=1.45; *P*=.15). The 200 most frequent words that appeared in the control and depressive users datasets are depicted in the 2 word clouds shown in Multimedia Appendix 1. The 10 most frequent words that appeared in the control dataset were the following: *hoy* (today), *día* (day), *ver* (to see), *quiero* (I want), *gracias* (thank you), *mejor* (better), *siempre* (always), *vida* (life), *ahora* (now), and YouTube. In the depressive users dataset, the 10 most frequent words were the following: *quiero* (I want), *vida* (life), *siempre* (always), *siento* (I feel), *nadie* (nobody), *mierda* (shit), *never* (nunca), and *día* (day). It should be noted that in the depressive tweets dataset, although there are several words in common with the depressive users dataset, we can find additional words that are not present in the other datasets, such as *vacío/a* (empty), *matar* (to kill), *desaparecer* (to disappear), *suicidar* (commit suicide), *muerta* (dead), *desastre* (disaster), *inútil* (useless,), *deprimida* (depressed as state in women), *depresiva* (depressed as a condition in women), and *insomnio* (insomnia). The word cloud of the depressive tweets dataset is shown in Multimedia Appendix 2. In relation to the use of links, hashtags, and mentions in tweets, the frequency of them in the control and depressive users datasets were 35.32% (251,728/712,584), 13.13% (93,575/712,588), and 44.00% (313,574/712,577) and 18.07% (25,475/140,946), 1.44% (2030/140,946), and 9.27% (13,060/140,942), respectively. The number of tweets, including emojis, were 13.61% (97,038/712,589) in the control dataset and 5.72% (8069/140,947) in the depressive users dataset.

**Table 2:** Part-of-Speech (POS) frequencies in tweets of control, depressive users, and depressive tweets datasets.

Type of POS	POS in the control dataset, n (%)	POS in the depressive users dataset, n (%)	POS in the depressive tweets dataset, n (%)
Noun	2,298,544 (28.48)	270,104 (17.77)	1776 (15.07)
Verb	1,660,700 (20.58)	400,755 (26.36)	3391 (28.77)
Pronouns	770,955 (9.55)	225,913 (14.86)	1627 (13.80)
Adjectives	593,327 (7.35)	83,089 (5.47)	588 (4.99)
Determiner	1,068,130 (13.23)	177,795 (11.70)	1342 (11.39)
Adverbs	496,988 (6.16)	140,963 (9.27)	1351 (11.46)
Adpositions	854,573 (10.59)	123,867 (8.15)	1052 (8.93)
Conjunctions	327,852 (4.06)	97,541 (6.42)	659 (5.59)

### Emotion Analysis

**Figure 5:**
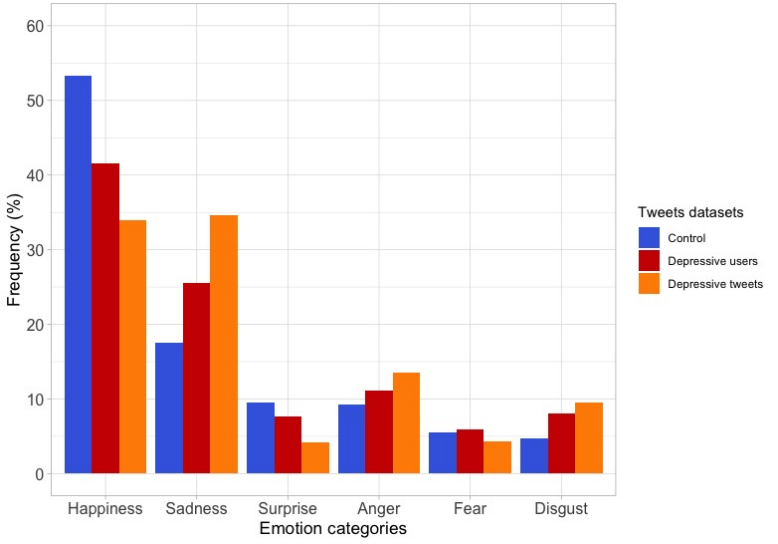
Frequency distributions of emotion categories in the tweets of the 3 datasets.

Regarding the distribution of emotions, in the control dataset and in the depressive users dataset, the most frequent emotion was happiness (53.30%; 203,029/380,874 and 41.60%; 40,535/97,425) followed by sadness, which was more frequent in the depressive users dataset (17.59%; 67,033/380,874 and 25.49%; 24,834/97,425). In the depressive tweets dataset, the most frequent emotion was sadness (34.00%; 303/891). There were statistically significant differences between the control and the depressive users datasets (χ^2^_5_=6838.2; *P*<.001), between the control and the depressive tweets datasets (χ^2^_5_=296.8; *P*<.001), and between the depressive users and the depressive tweets datasets (χ^2^_5_=65.6; *P*<.001). The frequencies of the different emotions are shown in Figure 5.

### Negation Words

Regarding the use of negation words, they were detected in 21.74% (154,953/712,588) of the tweets in the control dataset, in 34.15% (48,137/140,946) of the depressive users dataset, and in 45.50% (455/1000) of the depressive tweets dataset. The mean of negation words was 0.28 (SD 0.59) in the control dataset, it was 0.49 (SD 0.82) in the depressive users dataset, and it was 0.67 (SD 0.91) in the depressive tweets dataset. There were statistically significant differences between the control and the depressive users datasets (Mann-Whitney *U*=4.3657e+10; *P*<.001), between the control and the depressive tweets datasets (Mann-Whitney *U*=266,990,000; *P*<.001), and between the depressive users and the depressive tweets datasets (Mann-Whitney *U*=62,002,000; *P*<.001).

### Polarity Analysis

**Figure 6:**
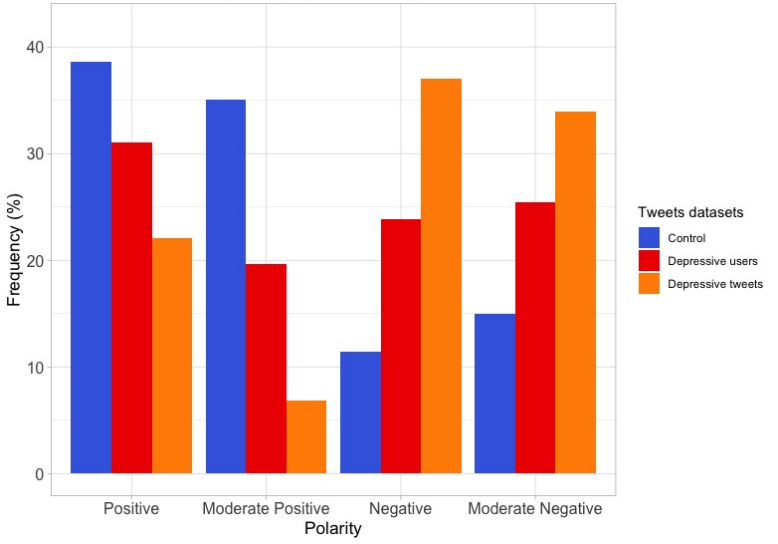
Polarities of the tweets according to the SentiCo Polarity tool in the 3 datasets.

In relation to the polarity of tweets, 2 analyses were performed using 2 Spanish sentiment lexicons: the Senti Lexicon (including positive and negative categories) and the SentiCo Polarity (including positive, moderate positive, moderate negative, and negative categories). According to the Senti Lexicon, the analysis of tweets showed that the control dataset shows polarity in 33.47% (245,367/733,029) of the tweets, being positive in 56,54% (138,726/245,367) of them. In contrast, the depressive users dataset shows polarity in 41,31% (61,132/147,996) of the tweets, being positive in 46.14% (28,205/61,132) of them. Finally, the depressive tweets dataset shows polarity in 58.90% (589/1000) of the tweets, with positive polarity in 34.97% (206/589) of them. There were statistically significant differences between the control and the depressive users datasets (χ^2^_1_=2134; *P*<.001), between the control and the depressive tweets datasets (χ^2^_1_=110.3; *P*<.001), and between the depressive users and the depressive tweets datasets (χ^2^_1_=28.8; *P*<.001). When using the SentiCo Polarity tool, the control dataset presented 20.97% (152,228/725,717) of tweets with polarity, 29.32% (42,820/146,033) in the depressive users and 33.34% (348/1,044) in the depressive tweets dataset. The distributions of polarities are shown in Figure 6. There were statistically significant differences between the control and the depressive users datasets (χ^2^_3_=8820.8, *P*<.001), between the control and the depressive tweets datasets (χ^2^_3_=308.8; *P*<.001), and between the depressive users and the depressive tweets datasets (χ^2^_3_=52.4; *P*<.001).

## Discussion

### Principal Findings

The diagnosis of depression is complex because of the heterogeneous nature of this disease and the diverse manifestation of the symptoms among individuals, which result in a great number of depressive disorder cases that are undetected and untreated, making the prevention, diagnosis, and treatment of the depressive disorders a complicated task [15,49,50]. For these reasons and taking into account that people diagnosed with depression are increasing worldwide, new strategies for detecting and monitoring this disease would be very useful. In this study, we analyzed the behavioral and linguistic patterns of tweets in Spanish that suggest signs of depression. The results contribute to the growing body of scientific literature that analyzes the messages posted on social media using languages other than English. We have introduced a new approach that comprises analyzing the timelines of self-qualified depressed users, as well as their tweets related to depression, which are manually selected. Our results show that the tweets of depressive users have different features in comparison with those of a control dataset, even when their tweets that are not related to depression are considered (depressive users dataset). In addition, the differences with the control dataset become more evident when we consider the manual selection of tweets related to depression (depressive tweets dataset).

### Different Distributions of Tweets Over Time

As for the distribution of tweets over time, the users of the depressive dataset, although being less active in using Twitter, used to be more active during the night than the users of the control dataset. This can be explained as a result of insomnia, one of the most frequent symptoms of depression. This finding is consistent with previous studies carried out with English speakers, which demonstrated that individuals with depression are more active during the night [20]. Moreover, the daily mood changes, such as the morning and evening worsening that are typical in several forms of depression, could explain the lower activity of the depressive users [51]. In relation to the distribution of tweets throughout the week, the users of the depressive dataset showed a more regular activity throughout the week, tending to be more active on Saturdays, Sundays, and Mondays than those of the control dataset, whose activity showed a drop during the weekend. This trend may be related to the lowered social activity of the people suffering from depressive disorders, having a reduced participation in social leisure activities during the weekend and spending more time at home, sharing their feelings and thoughts on social media platforms [16].

### Different Style of Writing

The analysis of POS and the number of words by grammatical categories show that, generally, the users of the depressive dataset used more verbs, adverbs, and pronouns but less nouns than the control dataset. The same features are also present in the depressive tweets dataset. These findings suggest that the language of people suffering from depression is characterized by a different style of writing that some authors describe as poorly structured, indicating less interest in what surrounds them, people, objects, or things [52]. They focus on talking about actions, and this is correlated with sensitive disclosure. Consistent with many previous studies [20,30,35,53-55], the use of first-person singular pronoun is more frequent among the users of the depressive dataset, with respect to those of the control dataset, and this difference increases in the depressive tweets dataset. The increased use of this pronoun demonstrates the attention to self-focus that is associated with the negative emotional states of depression and the reduced attentional resources, highlighting the psychological distancing to connect with others [56]. This social isolation may also explain that the first- and second-person plural pronouns are the least used. Language can be used as a measure of different individual features, on the basis of the fact that people’s word choice is stable over time and consistent across topics or context. For this reason, the language style appears to be a useful predictor of some mental health conditions, such as depression [20,35]. In addition, the number of characters written in the depressive users and depressive tweets datasets was smaller than the number of characters written in the control dataset, and this might be related to reduced interest and poorer language. According to the most frequent words that appeared in the depressive users and depressive tweets datasets, there are specific words that are linked to clinical symptoms and the way that depressive patients word their mood, such as words that may be related to suicide ideation. Consequently, they can be used as a signal to detect potentially depressed users on Twitter [36]. Similarly, we observed the frequent use of adjectives in feminine form in the depressive tweets dataset, which would suggest that a high proportion of the depressive users are women, a fact that is in agreement with clinical and epidemiological evidences [8,11,12,42].

### Predominant Emotions

Emotions are one of the key aspects that characterize many mental health conditions and, particularly, when people are suffering from depression. An analysis of the 6 emotions that are commonly considered (happiness, sadness, surprise, anger, fear, and disgust) [47] was performed to determine the existence of differences among the datasets. Happiness is the most frequent emotion in the control and depressive users datasets, although an important reduction was observed in the depressive tweets dataset. The surprise emotion is less frequent in depressive users and, specially, in the depressed tweets datasets than the control dataset, and this fact can be related to the depressive mood, in which there is a decrease in interest in almost everything. Fear does not seem to be a differential emotion in the groups of tweets analyzed in this study.

Regarding negative emotions, we observed an increase in the frequency of words related to the sadness emotion in the depressive tweets dataset, doubling that of the control dataset. This feature had also been observed in other studies [14,35,57]. Moreover, anger is more frequent in the depressive user and depressive tweets datasets than in the control dataset. Although Twitter is used many times for expressing anger about personal or political aspects, this emotion is particularly frequent in patients suffering from depression, who tend to feel irritable, wronged, or angry at the world [14,16,35,58]. At the same time, disgust, an emotion that is known to be associated with the depressive disorders [59], was found to be more frequent in the depressive users and depressive tweets datasets.

### Negative Focused Emotion Language

In our analysis, the presence of negation words is more frequent in the depressed users (34.15%; 48,137/140,946) and depressive tweets (45.50%; 455/1000) datasets than in the control dataset (21.74%; 154,953/712,588), indicating that there is an increased use of negatively focused emotion language, which is typical in depressive patients and feelings [31,54,55,60].

### Negative Polarity

The classification of tweets, on the basis of the emotional positivity or negativity of their words, is another analysis that has been carried out. In this study, we used 2 types of polarity lexicons, the Senti Lexicon (SentiLex) and the Sentico Polarity (SentiCo). In both cases, the negative polarity was higher in the depressive users and depressive tweets datasets, even tripling the negativeness of the control dataset when using the Sentico Polarity lexicon. These findings are consistent with other studies, indicating that people suffering from depression tend to focus more on negative aspects of their life [20,35], and thus their tweets contain much more negative emotional words compared with the control dataset [14]. In addition, the self-focus state that characterizes depression is associated with negative emotions [32,56,57].

### Limitations and Future Directions

This study presents some limitations that have to be pointed out. On the one hand, the tweets of the depressive datasets come only from Twitter users who speak publicly about feelings and emotions that can be related with depression. This is an indirect and inaccurate way of detecting users suffering from depressive disorders. Without clinically assessing these people, there is no way to verify if the diagnosis is genuine or if they suffer from another mental disorder. On the other hand, it is possible that Twitter users self-disclose their mental health using words or expressions not included in the list of keywords used in this study for streaming tweets about depression [22,61-63]. In this respect, it is possible that a wider list could have yielded a greater coverage [21,36]. Privacy policies of social media restrict the access to users who did not grant access to their profile, and this may have generated biases in the composition of the depressive users and the depressive tweets datasets. In addition, tweets may incorporate biases because of the self-management and anonymity of the Web-based identities [61]. Moreover, Twitter users may be not be representative of the general population, and some studies have shown that they are often urban people with high levels of education [64-66]. More information about the socioeconomic and demographic details of Twitter users is needed [67]. The control dataset was a randomly selected sample of Twitter users, and it is consequently representative of the users of this social media. However, there is a possibility that users in this group may also have depression or other mental illness even though they did not mention this in their profile description. There is also the possibility that the users included in the control group are fake accounts. Only original tweets were analyzed, and perhaps retweets, which are not included in our linguistic study, reflect users’ emotions that can be related to depression status [68]. Finally, depression is a very complex mental disorder, and our study only provides a general observation of this disorder. Additional research might be carried out to examine specific depression types and determine if there are social media features that can contribute to classifying users or tweets to the different diagnosis of depression [69]. Similarly, in future works, we plan to study the linguistic features and the behavioral patterns of depression in different linguistics contexts. The possible relationship between depression and seasonality could be of interest for future studies in the context of monitoring Twitter activity [70].

### Conclusions

The prevalence of common mental disorders worldwide, such as depression, requires the ability of health care systems to provide adequate diagnosis, monitoring, and treatment. The wide popularity of social media platforms introduces new opportunities for the screening of depression. The introduction of new methods of analysis for the automatic detection of signals of depression on social media platforms, such as Twitter or Facebook, has the potential of being used as a complementary tool for the assessment of these patients, assisting health care professionals in the detection and monitoring of mental health disorders. Although the analysis of tweets as a way to determine the existence of depression cannot be used as a replacement for diagnosis, it has the potential as a screening tool for depressive disorders, with a lower cost than other traditional procedures. In addition, it can be helpful to health professionals for managing and monitoring patients more efficiently. Similarly, it can be useful for particular patients, as they feel more comfortable disclosing their symptoms on Twitter than in clinical settings. In this study, we have shown that several behavioral and linguistic features of the tweets in Spanish can be used as a complementary tool to detect signals of depression of their authors, corroborating and extending the findings obtained by studies carried out on English tweets. As we described in this study, signs of depression of Twitter users are not exclusively spotted by identifying and analyzing tweets that explicitly mention expressions related to depression. Moreover, Twitter users who are potentially suffering from depression globally modify the core traits of their language, independently from the fact that the tweets are related or not related to the expression of depression. On the basis of these changes, these users can be monitored and supported. The results of this paper, jointly with other studies on the matter, support the potential of social media as an important instrument for extending and enhancing mental health services available to people with mental disorders. By means of interdisciplinary collaborations, it is possible to develop digital apps and services providing personalized alerts and psychosocial support in the mental health domain.

## Abbreviations

API: Application Programming Interface
POS: Part-of-Speech
WHO: World Health Organization

## Appendix

Multimedia Appendix 1 Word clouds showing the 200 most frequent words in the control (left) and depressive users (right) datasets.

Multimedia Appendix 2 Word cloud showing the 200 most frequent words in the depressive tweets dataset.
