# Supplementary figures and images for: Detecting Signs of Depression in Tweets in Spanish: Behavioral and Linguistic Analysis
Source: J Med Internet Res. 2019 Jun 27;21(6):e14199. doi: 10.2196/14199 (PMC6620890; doi:10.2196/14199)

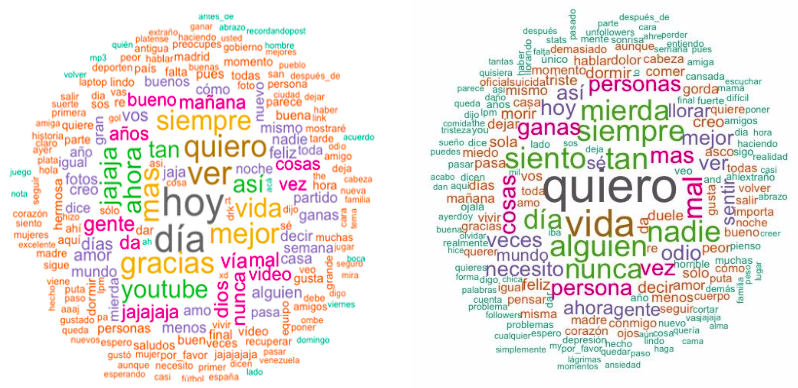

Supplement: Multimedia Appendix 1 [file jmir_v21i6e14199_app1.png]

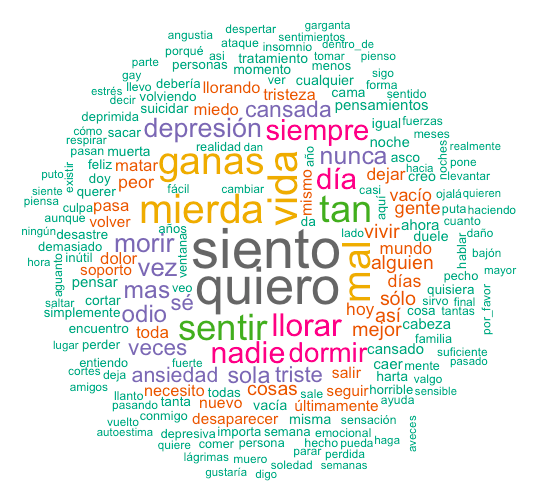

Supplement: Multimedia Appendix 2 [file jmir_v21i6e14199_app2.png]
